# Study of the diagnostic accuracy of microbiological techniques in the diagnosis of malaria in the immigrant population in Madrid

**DOI:** 10.1186/s12936-018-2459-2

**Published:** 2018-08-29

**Authors:** Ariadna Martín-Díaz, José Miguel Rubio, Juan María Herrero-Martínez, Manolo Lizasoain, José Manuel Ruiz-Giardin, Jerónimo Jaqueti, Juan Cuadros, Gerardo Rojo-Marcos, Pablo Martín-Rabadán, María Calderón, Carolina Campelo, María Velasco, Ana Pérez-Ayala

**Affiliations:** 1 Department of Clinical Microbiology, Hospital Universitario 12 de Octubre, Avenida de Córdoba, s/n, 28041 Madrid, Spain; 2 Malaria & Emerging Parasitic Diseases Laboratory, Parasitology Department, National Microbiology Centre, Instituto de Salud Carlos III, Madrid, Spain; 3 Internal Medicine and Infectious Diseases Department, Hospital Universitario 12 de Octubre, Madrid, Spain; 4 University Hospital of Fuenlabrada, Madrid, Spain; 5 University Hospital Príncipe de Asturias, Alcalá de Henares, Madrid, Spain; 6 University Hospital Gregorio Marañón, Madrid, Spain; 7 University Hospital Fundación Alcorcón, Madrid, Spain

**Keywords:** Submicroscopic parasitaemia, PCR *Plasmodium*, Malaria diagnosis

## Abstract

**Background:**

Malaria is currently the most important human parasitic disease in the world responsible for high morbidity and mortality. Appropriate diagnostic methods are essential for early detection. Microscopy examination remains the *gold standard*, although molecular techniques have higher sensitivity and are very useful in cases of low parasitaemia and mixed infections. The objective of this study was to evaluate a new commercial molecular diagnostic technique.

**Methods:**

A prospective, observational, multicentre study was performed between January 2015 and April 2017. All participants were immigrants from malaria-endemic areas, who were divided into two groups: asymptomatic group and symptomatic. Samples from both groups were evaluated by a rapid diagnostic test (ImmunoQuick^®^ Malaria + 4 RDT), microscopy examination, and two commercial molecular malaria tests (FTD Malaria and FTD Malaria Differentiation), then compared against an in-house reference PCR technique.

**Results:**

In all, 250 patients were included: 164 (65.6%) in the asymptomatic group, and 86 (34.4%) in the symptomatic group. There were seven cases of asymptomatic parasitaemia (prevalence = 2.8%) that were detected only by molecular methods. In the symptomatic group, there were seven cases of submicroscopic malaria. The main species detected was *Plasmodium falciparum* (96.6%). The commercial molecular technique had higher sensitivity than the other methods (S = 96%) and a high rate of concordance with the in-house reference PCR technique (Kappa score = 0.93).

**Conclusions:**

The molecular techniques, although slower than microscopy, have adequate diagnostic accuracy and are very useful for the detection of *P. falciparum* in cases with low parasitaemia.

## Background

Malaria is an overwhelming problem in tropical developing countries. It is currently the most important human parasitic disease in the world and continues to be a serious public health problem [1]. More than 85% of malaria cases and 90% of malaria deaths occur in sub-Saharan Africa, mainly in young children (especially those under 5 years old) [2].

Effective diagnostic techniques permit early and appropriate diagnosis of malaria since this prevents the infection from progressing to a severe stage or even death. The World Health Organization (WHO) recommends confirming all clinically suspected malaria cases before treatment using a malaria-specific rapid diagnostic test (RDT) or by direct visualization of the parasites using standard microscopy [3, 4].

Light microscopy requires a high level of expertise, involves examining Giemsa-stained thick and thin blood smears on glass slides. Thick blood smears remains the clinical *gold standard* method for detecting *Plasmodium* in blood. With this technique, under optimal conditions, an experienced microscopist can achieve a sensitivity of about 50 parasites/µL of blood, although the sensitivity of detection in most routine diagnostic laboratories is normally much lower (in the range of approximately 50–100 parasites per µL of blood) [1, 5]. This method, also examining thin blood smears, allows for species identification and quantification of parasitaemia, but it is a time-consuming technique for the detection of scanty parasites and is often difficult to use accurately for the identification of mixed infections.

RDTs are lateral flow immunochromatographic devices that detect *Plasmodium* antigens, such as histidine-rich protein 2 (HRP2), produced by *Plasmodium falciparum*, parasite-specific lactate dehydrogenase (pLDH) or aldolase enzyme, which is common to all species. Currently available RDTs have lower sensitivity than well-trained microscopists (≈ 100 parasites/µL) [1] and are of limited use [6] in cases with parasite densities below the threshold of microscopy detection. The results of WHO product testing of malaria RDTs round seven reported that a number of RDTs consistently detected malaria at low parasite densities (200 parasites/µL), had low false-positive rates and could detect *P. falciparum*, *Plasmodium vivax* or both of them [7].

Molecular techniques have been designed to minimize the problems associated with the other methods described above [5]. They are highly sensitive and can detect ≤ 5 parasites/µL of blood (even parasitaemias as low as one gene copy per reaction). A significant proportion of malaria infections is associated with a submicroscopic parasitaemia whose clinical profiles can only be routinely detected using polymerase chain reaction (PCR)-based techniques [1, 8] rather than conventional microscopy or RDTs. PCR is also very helpful for identifying malaria cases caused by more than one species, which are sometimes difficult to identify by microscopy. To date, the only available molecular techniques are in-house PCR, only performed at Reference Centres.

The main objective of this study was to evaluate a new commercial molecular technique by comparing it with other diagnostic methods: thick and thin blood film examination, RDT and an in-house reference PCR technique, in two different population groups.

## Methods

A prospective, observational, multicentre study was performed. Data were collected at five different hospitals in Madrid, Spain (University Hospital 12 de Octubre; University Hospital of Fuenlabrada, University Hospital Príncipe de Asturias, University Hospital Gregorio Marañón, and the University Hospital Fundación Alcorcón) between January 2015 and April 2017.

A 5 mL blood sample was collected from all participants. All the samples were tested using all the diagnostic techniques: RDT (ImmunoQuick^®^ Malaria + 4, Biosynex), Giemsa-stained thin and thick blood films, the commercial molecular technique (C-PCR) under evaluation, and the reference molecular technique (CNM-PCR).

The ImmunoQuick^®^ Malaria + 4 RDT is a lateral flow immunochromatographic method that detects the presence of *P. falciparum*-specific histidine-rich protein-2 (Pf HRP-2) and pan malaria-specific pLDH (pan lactate dehydrogenase) in human blood. The tests were performed by a microbiologist at the different hospitals, in accordance with the manufacturer’s instructions.

Thin and thick blood films were stained with Giemsa solution (1:10 dilution) for 10 min. All slides were examined under a light microscope with oil-immersion objectives (100×) at the Hospital 12 de Octubre. The same person, a well-trained microscopist blinded to the results of the other tests, carried out the examination in at least 200 visual fields. If the sample was positive, parasitaemia was calculated, and the result expressed in percentage form as the number of parasitized red blood cells.

CNM PCR was performed at the National Centre of Microbiology (Instituto de Salud Carlos III) by the same person, an expert microbiologist blinded to the results of the other tests. This technique allows for identification of the four major malaria species in human blood. The method is a semi-nested multiplex PCR involving two multiplex PCR reactions for amplification of target sequences. The first reaction amplifies *Plasmodium* ssrDNA to detect *Plasmodium* in blood samples and includes an internal amplification reaction control. The second reaction, an identification reaction, is performed to enable *Plasmodium* species identification [5, 9]. DNA extraction from 200 µL of whole blood was performed using the QIAamp DNA Blood Mini Kit, following the manufacturer’s instructions, with elution volumes of 200 µL using the QIAcube System (QIAGEN, Hilden, Germany). The cost of the Nested Multiplex PCR is around one and a half euro per reaction plus the DNA isolation (5 euros).

The same blinded microbiologist who examined blood films also performed C-PCR at the Hospital 12 de Octubre. DNA extraction from 400 µL of venous blood was carried out with RBC Bioscience’s MagCore^®^ Compact Automated Nucleic Acid Extractor, and MagCore^®^ Genomic DNA Whole Blood Kit 102 extraction cartridges. All samples were extracted with an internal control to ensure the validity of the extraction and amplification processes. Purified DNA was eluted in a final volume of 60 µL, which was maintained stored at − 20 °C until amplification. This PCR method (FTD Malaria, Fast-Track Diagnostics^®^, Esch-sur-Alzette, Luxembourg) consists of two different reagent kits: a generic PCR test for detection of *Plasmodium* spp, and a differential PCR reagent kit, able to detect four species of *Plasmodium* (*P. falciparum*, *P. malariae*, *P. ovale* and *P. vivax*). *Plasmodium knowlesi* is not identified by the differential PCR but it would be detected by the first PCR as *Plasmodium* spp. In both PCRs 10 µL of extracted DNA are used, as well as for negative and positive controls. The test consists of two processes in a single tube assay: PCR amplification of target DNA and Internal Control, and simultaneous detection of PCR amplicons by fluorescent dye labelled probes. Both reactions were performed using the CFX96™ Real-Time System, BioRad^®^thermal cycler. All samples were analysed by generic PCR, and positive samples were further analysed by differential PCR. The cost of the *Plasmodium* spp. PCR is 22.5 euros per reaction (DNA isolation included), if a positive result is obtained 19 euros have to be added to know the species.

C-PCR is a real time PCR which only needs one amplification reaction and it can be performed at the same hospital, so it can be done faster and with less contamination risk than the CNM PCR. A formula can directly be used to quantify Ct values of differential PCR, so results can also be reported as gene copy numbers/mL.

Participants were divided into two groups: asymptomatic and symptomatic group. The asymptomatic group included immigrants over 18 years of age originating from malaria-endemic areas (sub-Saharan Africa, Southeast Asia, the Indian subcontinent, Haiti and areas of the Amazon rainforest in South America), who presented at the hospital without fever or initial suspicion of malaria. In these cases, molecular techniques were expected to detect some submicroscopic carriers. Patients who fell into this category were located in order to offer them malaria treatment.

The symptomatic group included all patients who attended one of the participant hospitals with symptoms suggestive of malaria and parasites in at least one test.

The study was carried out following the ethical principles of the Helsinki Declaration. Before the samples were collected, participants were provided with an explanation of the nature of the project and given an informed consent form, which they signed. Ethical review and clearance were obtained from the Medical and Health Research Ethics Committee at each of the hospitals participating in the project.

### Statistical analysis

All variables were included in an online database “REDCap” (Research Electronic Data Capture) previously created for the study. The Kappa statistic and efficiency index were used to compare and rate RDT, microscopy and C-PCR versus the reference method, CNM-PCR. Diagnostic parameters such as sensitivity, specificity, positive predictive value, negative predictive value, positive likelihood ratio, negative likelihood ratio and efficiency were calculated for each diagnostic method. All estimates were reported with 95% confidence intervals (CI). Quantitative variables were expressed as means and standard deviation (SD) or as median (25th–75th percentiles). Categorical variables were expressed as frequencies. Relationships between categorical variables were compared using the χ^2^ test. For relationships between quantitative and categorical variables, the Fisher Student’s *t* test or the Mann–Whitney U test were used as appropriate. Statistical significance was set at *p *< *0.05*. Data were analysed using SPSS version 18.0 (SPSS Inc, Chicago, IL, EEUU) and EpiDat 3.1 software version (Consellería de Sanidade, Dirección Xeral de Saúde Pública, Xunta de Galicia, Spain).

## Results

**Fig. 1:**
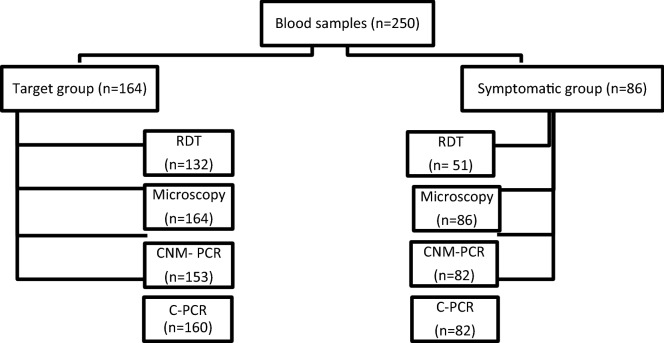
Distribution of blood samples collected according to each group of patients

During the study period, 250 patients in all were included: 164 (65.6%) in the asymptomatic group and 86 (34.4%) in the symptomatic group. All 250 blood samples were analysed by microscopy, 184 by the RDT (132 and 52 in the target and symptomatic groups, respectively), 235 by CNM-PCR (153 and 82 in the target and symptomatic groups, respectively), and 242 by C-PCR (160 and 82 in target and symptomatic groups, respectively). Fifteen patients did not have enough blood to perform CNM-PCR. C-PCR could not be performed on eight patients for the same reason (Fig. 1).

The *Immunoquick*^*®*^ *Malaria* + *4* test gave a negative result for most of the patients in the asymptomatic group. A positive result was observed in only three cases. Two of these were false positives, which were confirmed by negative microscopy and negative PCR, and the other was a true positive. By contrast, in the symptomatic group, this method gave two false negative results.

In the symptomatic group, the microscopic examination was positive in 79 patients (91.8%). There were seven cases of symptomatic patients with a negative microscopy, but where molecular methods detected *Plasmodium*. Infection was confirmed in five cases by both PCR methods (CNM-PCR and C-PCR) and the other two were detected only by C-PCR. Six cases were due to *P. falciparum* and the other one was a mixed infection due to *P. falciparum, Plasmodium ovale* and *Plasmodium malariae*. All but one had come from Equatorial Guinea.

Microscopy was negative in all patients in the asymptomatic group, with the exception of one patient from Nigeria, who had arrived in Spain 42 days before. Seven cases were detected only by molecular techniques: one case was diagnosed by both CNM-PCR and C-PCR, three only by CNM-PCR, and three only by C-PCR. The prevalence of asymptomatic parasitaemia was 2.8%. All were *P. falciparum* infections and were detected in patients coming from sub-Saharan Africa.

Overall, there were 90 cases of malaria. The main species detected by PCR was *P. falciparum* (87/90: 96.6%): 8 in the asymptomatic group, 79 in the symptomatic group. Other species were detected only in the symptomatic group. There were four cases of *P. malariae* and all were mixed infections*. Plasmodium vivax* was detected in three samples by PCR: in two cases it was the only species detected, and the other one was a mixed infection with *P. malariae*. There were eight cases of *P. ovale* and all but one were mixed infections, as specified in Table 1. The two patients with *P. vivax* infection originated from India and the mixed infection with *P. malariae* were from Equatorial Guinea.

**Table 1:** Species detected in the asymptomatic and symptomatic groups of patients

Detected species (n = number of cases)	Asymptomatic group	Symptomatic group
*Plasmodium falciparum* (n = 87)	8	79
*Plasmodium malariae* (n = 4)	0	4^a^
*Plasmodium vivax* (n = 3)	0	3^b^
*Plasmodium ovale* (n = 8)	0	8^c^

^a^All were mixed infections: two were *P. malariae *+ *P. falciparum*, one *P. malariae *+ *P. vivax* and one *P. malariae *+ *P. falciparum *+ *P. ovale*

^b^One of these was a mixed infection with *P. malariae*

^c^Seven cases were mixed infections: six with *P. falciparum,* one with *P. falciparum *+ *P. malariae *+ *P. ovale*

There were 10 cases of mixed infections (11.1%), all in the symptomatic group. Six cases were due to *P. falciparum* together with *P. ovale*, two to *P. falciparum* and *P. malariae*, and one to *P. vivax* and *P. malariae*. One infection was caused by three species (*P. falciparum*, *P. malariae* and *P. ovale*). Eight cases showed discrepancies between the results obtained by CNM-PCR and C-PCR.

The diagnostic rates obtained with each of the different techniques in this study were then compared with those of the reference, CNM-PCR (Table 2). A total of 178 blood samples were analysed by both CNM-PCR and RDT, 234 by both CNM-PCR and microscopy (thick blood slide), and 228 by both CNM-PCR and C-PCR.

**Table 2:** A summary of results of the different diagnostic parameters compared to CNM-PCR

Global, (n)	Sensitivity (95% CI)	Specificity (95% CI)	PPV (95% CI)	NPV (95% CI)	PLR (95% CI)	NLR (95% CI)	Efficiency (95% CI)	Kappa score (95% CI)
RDT (n = 178)	88.88 (76.50–95.16)	84.21 (77.06–89.43)	95.24 (87.61–100)	97.01 (93.76–100)	60 (15.12–238.14)	0.09 (0.04–0.24)	96.5 (93.63–99.56)	0.65 (0.53–0.77)
Thick blood slide, (n = 234)	88.46 (79.50–93.80)	100 (97.59–100)	100 (99.28–100)	94.55 (90.78–98.31)	–	0.12 (0.06–0.21)	96.59 (93.86–99.32)	0.91 (0.85–0.96)
C-PCR, (n = 228)	96.00 (88.88–98.63)	97.38 (93.47–98.97)	93.59 (87.51–99.67)	98.04 (95.52–100)	29.78 (12.56–70.61)	0.04 (0.01–0.12)	96.97 (94.33–99.61)	0.93 (0.88–0.98)

*PPV* positive predictive value, *NPV* negative predictive value, *PLR* positive likelihood ratio, *NLR* negative likelihood ratio

Overall, when compared with microscopy, the molecular techniques resulted in an increase in the detection of infection, with high concordance (Kappa score 0.91) between microscopic examination of thick blood slides and PCR. There was a high concordance between the two molecular methods, showing a Kappa score of 0.93. C-PCR was able to detect more cases of malaria infection, as well as mixed infections.

## Discussion

It is very important to make a definitive diagnosis and confirm malaria disease. Although microscopy remains the *gold standard* for malaria diagnosis and is faster than molecular techniques, it has major limitations such as low sensitivity, difficulties associated with quality control and standardization and the need for ongoing training and evaluation [10–13]. For this reason, PCR assays are increasingly being used in the diagnosis of malaria [3, 10, 14, 15]. Based on published findings, studies have demonstrated that PCR methods are more sensitive and allow for more accurate species identification than microscopy [10, 15, 16]. On the other hand they require well-trained staff and time to obtain a result is longer than for microscopy (around 3 h compared to no more than 1 h for microscopy).

As a result of globalization, the number of imported malaria cases has increased, mainly due to immigrants from malaria-endemic areas who have developed a semi-immune status [17–19], which they gradually lose when they live outside their home country. As a result, individuals in this population are at a high risk of contracting malaria when they travel, they tend to have low-density parasitaemia, and may be misdiagnosed if only microscopy and RDTs are employed [4]. The presence of these asymptomatic carriers could be a risk in countries where malaria is not endemic, but where there are vectors, as in Spain, which is a malaria-free country with anophelism (*Anopheles atroparvus*).

For all these reasons, a new commercial technique was evaluated and its usefulness in routine malaria diagnosis by comparing it with a RDT, microscopy, and a reference PCR technique. These data show that microscopy gives a more accurate diagnosis than the dipstick RDT ImmunoQuick^®^ Malaria + 4 RDT, but has lower diagnostic capacity (sensitivity 88.5%) than PCR. More specifically, 14 out of 90 patients had a negative microscopy and were diagnosed only by PCR. The thick blood slide examination was positive in only one patient with very low parasitaemia (< 0.01%) in the asymptomatic group. In malaria infections where parasitaemia is very low, diagnosis can easily be missed if it is not possible to use a molecular technique and expert microscopists are not available.

The sensitivity and specificity of RDT ImmunoQuick^®^ Malaria + 4 RDT were both less than 90%, with a Kappa score of 0.65 against CNM-PCR. Its usefulness in malaria diagnosis lies mainly in the fact that it is quick and easy to perform and does not require trained staff. A product evaluation by the manufacturer, reporting findings from 230 samples, showed that the sensitivity and specificity of Immunoquick^®^ Malaria + 4 versus microscopic examination of thick and thin blood smears were 70.5% and 100% for pLDH, and 98.8% and 100% for Pf HRP-2, respectively. In the present study, sensitivity and specificity were 88.8% and 84.2%, respectively, lower than those specified by the manufacturer. In the asymptomatic group, the RDT yielded two false positive results, confirmed by negative microscopy and negative PCR. This test also gives false negative results in patients with low parasite densities. Of the seven cases of asymptomatic parasitaemia, the RDT was positive in only one sample; on the other hand, the RDT detected all cases of symptomatic submicroscopic malaria except one. In *P. falciparum* malaria infection, HRP-2 is not secreted in the gametogony stage, so that the HRP-2 band may be absent in healthy carriers and may be overlooked.

C-PCR is easy to perform and it can be done fast at the same hospital. Also, it is standardized and has good diagnostic parameters (sensitivity 96%, specificity 97.38%, Kappa score 0.93), which were comparable with those of CNM-PCR. Among submicroscopic infections, eight discrepant results were found between the two PCR techniques: two in the symptomatic group and six in the asymptomatic group. The discrepancies in the asymptomatic group could be due to low parasitaemia levels. The two discrepant results in the symptomatic group were detected only by RDT and C-PCR, whereas microscopy and CNM-PCR were negative. It is normal to find some variability in sensitivity scores between different PCR methods [20], attributable to the intrinsic variability of the technique, the specific target detected by the method, and differences in the extraction process (sample volume, elution volume). This is particularly important in mixed infections, since, although both molecular methods were able to detect at least one species, there were discrepancies in eight cases. If only CNM-PCR or C-PCR had been performed, 5 and 2 of them, respectively, may have gone unnoticed.

After considering the results obtained at the study centre, the authors conclude that molecular methods show improved sensitivity over microscopy, mainly in asymptomatic parasitaemias. The combination of microscopy and PCR upgraded the diagnosis of malaria, as the sensitivity, specificity, VPP and VPN data demonstrate. Nevertheless, PCR also has its disadvantages, since it detects non-viable parasites after malaria episodes that have been correctly treated and this may lead to false positive results and unnecessary malaria treatments. In addition, it requires a sophisticated laboratory setting and trained technicians, requires a longer time to diagnosis and incurs higher costs [4]. For these reasons, probably none of these PCRs will be suitable enough to be included in the routine management at most malaria endemic settings. Other nucleic acid amplification techniques are currently emerging, such as loop-mediated isothermal amplification (LAMP), which has comparable sensitivity to nested PCR and has also reduced assay times (to within 1 h) [21–23]. It is easy to use, since it needs only a compact incubator/reader that requires minimal training for laboratory technicians [21] and could be a potentially useful alternative mainly in endemic areas, to current molecular tools, which are more complex [24].

*Plasmodium falciparum* was the most prevalent species diagnosed (96.6%). A low prevalence of malaria infections due to non-*falciparum* species was found, so that it was not possible to assess the diagnostic capacity of the methods in these cases. Mixed infections were not very frequent. Co-infections were detected in only 10 cases, all of them in the symptomatic group. Among co-infections, there were more discrepancies detected between the results obtained by CNM-PCR and C-PCR. Only two cases of mixed infections were detected by the two methods.

The prevalence of submicroscopic parasitaemia in the asymptomatic group (2.8%) was in line with data published by Monge-Maillo et al. [25] and Matisz et al. [26], but was lower than that reported in others [17, 27]. Seven cases of asymptomatic parasitaemia were diagnosed only by molecular methods; all were due to *P. falciparum*. The two molecular methods were positive in only one case. It is important to bear in mind that some cases may go undetected due to variability between methods. Higher sensitivity would be desirable to ensure a proper diagnosis and prompt treatment of malaria infection.

The study has some underlying limitations. It was not possible to perform all the diagnostic tests on all samples for various reasons. First, factors associated with this group of immigrant patients (difficult social situation, legal problems, language/cultural barrier) made it difficult to include them in the study. Second, in most cases of malaria infection, the species responsible was *P. falciparum*, so that the usefulness of the methods for non-*falciparum* species was not possible to assess. Third, there were too few cases of mixed infections to evaluate the usefulness of these methods for diagnosis. In this type of infection, one of the species involved is usually present in a smaller proportion and cannot be detected. Further studies are required to assess this problem.

## Conclusions

The FTD-Malaria and Malaria Differentiation PCRs showed good test values for the diagnostic test parameters (sensitivity, specificity, positive and negative predictive values and likelihood ratios), as well as good inter-procedure agreement for detecting malaria infections.

The population of immigrants originating from malaria-endemic areas can have low parasite blood densities that non-molecular methods fail to detect. PCR is a useful tool for early detection of this type of infection. In this study, the combination of two molecular techniques enabled 14 cases (1.6%) of submicroscopic infection to be detected. Molecular techniques are also the most reliable tests to make a correct diagnosis of *Plasmodium* species and mixed infections that, due to the subjectivity of microscopy, can be misdiagnosed.

The data obtained in this study are in agreement with other published reports and suggest that, while microscopy is very useful and faster, PCR is more sensitive and improves the diagnosis of malaria, above all in patients with undetectable parasitaemia. These data show that PCR is a diagnostic tool with efficiency, sensitivity and specificity greater than 96% for the diagnosis of malaria infections. Further research should be carried out to evaluate the usefulness of PCR and assess the real prevalence of submicroscopic infections.
